# COP1 Mediates Dark-Induced Stomatal Closure by Suppressing *FT*, *TSF* and *SOC1* Expression to Promote NO Accumulation in Arabidopsis Guard Cells

**DOI:** 10.3390/ijms232315037

**Published:** 2022-11-30

**Authors:** Yu-Yan An, Jing Li, Yu-Xin Feng, Zhi-Mao Sun, Zhong-Qi Li, Xiao-Ting Wang, Mei-Xiang Zhang, Jun-Min He

**Affiliations:** College of Life Sciences, Shaanxi Normal University, Xi’an 710119, China

**Keywords:** *Arabidopsis thaliana*, COP1, darkness, FT, guard cell signaling, hydrogen peroxide, nitric oxide, SOC1, TSF

## Abstract

RING-finger-type ubiquitin E3 ligase Constitutively Photomorphogenic 1 (COP1) and floral integrators such as FLOWERING LOCUS T (FT), TWIN SISTER OF FT (TSF) and SUPPRESSOR OF OVEREXPRESSION OF CONSTANS1 (SOC1) have been identified as regulators of stomatal movement. However, little is known about their roles and relationship in dark-induced stomatal closure. Here, we demonstrated that COP1 is required for dark-induced stomatal closure using *cop1* mutant. The *cop1* mutant closed stomata in response to exogenous nitric oxide (NO) but not hydrogen peroxide (H_2_O_2_), and H_2_O_2_ but not NO accumulated in *cop1* in darkness, further indicating that COP1 acts downstream of H_2_O_2_ and upstream of NO in dark-induced stomatal closure. Expression of *FT*, *TSF* and *SOC1* in wild-type (WT) plants decreased significantly with dark duration time, but this process was blocked in *cop1*. Furthermore, *ft*, *tsf*, and *soc1* mutants accumulated NO and closed stomata faster than WT plants in response to darkness. Altogether, our results indicate that COP1 transduces H_2_O_2_ signaling, promotes NO accumulation in guard cells by suppressing *FT*, *TSF* and *SOC1* expression, and consequently leads to stomatal closure in darkness. These findings add new insights into the mechanisms of dark-induced stomatal closure.

## 1. Introduction

Plant guard cells open and close stomata to regulate CO_2_ uptake for photosynthesis and control water loss from the plant. They are sensitive to multiple internal and external signals to set the appropriate stomatal aperture for the prevailing environments [1]. Light/dark is one of the most essential and well-studied environmental signals regulating stomatal movement [2,3,4]. Stomata open in response to light and close in the dark. Various components in light/dark-regulated stomatal movement, especially in light signaling, have been demonstrated, such as photoreceptors, plasma membrane H^+^-ATPase, ion channels, Constitutively Photomorphogenic 1 (COP1), CONSTANS (CO), hydrogen peroxide (H_2_O_2_) and nitric oxide (NO) [2,5,6,7].

COP1, an E3 ubiquitin ligase, has been extensively studied as a major negative regulator of photomorphogenesis [8]. Mao et al. [6] found that blue light-induced stomatal opening by cryptochromes (CRY) was mediated by negatively regulating COP1, first defining a new role of COP1 in guard cell signaling. After that, increasing evidence demonstrates that COP1 plays an essential role in modulating stomatal movement. Wang et al. [2] showed that COP1 acted downstream of Phytochrome B (PHYB) in regulating red light-induced stomatal opening. Several researchers further demonstrated that COP1 is not only involved in the light-induced stomatal opening but also plays a fundamental role in abscisic acid (ABA)- [9,10,11], dehydration- [12], and ultraviolet-B (UV-B)-induced stomatal closure [13]. These reports confirm the importance of COP1 in guard cell signaling. However, the underlying mechanisms are still largely unknown. In addition, although COP1 has been indicated to repress stomatal opening in darkness [2,6], its role in dark-induced stomatal closure and the components in COP1-mediated guard cell signaling in darkness has yet to be further elucidated.

COP1 represses plant photomorphogenesis by promoting the ubiquitin-mediated protein degradation of positive regulators, including light-signaling transcription factor long hypocotyl 5 (HY5) and CO [8]. Ge et al. [13] found that HY5 was required for COP1 signaling in UV-B-induced stomatal closure. Therefore, we wonder whether COP1 function in dark-induced stomatal closure is also through the HY5 route. FLOWERING LOCUS T (FT), TWIN SISTER OF FT (TSF), the closest homolog of FT, and SUPPRESSOR OF OVEREXPRESSION OF CONSTANS1 (SOC1) are well-known major regulators of flowering response downstream of the transcriptional regulator CO [8,14,15]. Additionally, these floral integrators have also been identified as general growth regulators in diverse other developmental processes, including fruit set, vegetative growth and stomatal control [5,14,15]. Kinoshita et al. [16] demonstrated that FT played a role in regulating blue-light-induced stomatal opening by activating the plasma membrane H^+^-ATPase. Later, TSF and SOC1 were also proved to express in guard cells and exert positive effects on stomatal opening [5,17]. These studies defined a novel function for these floral integrators in stomatal opening. However, it is still unknown whether FT, TSF and SOC1 are involved in COP1-mediated darkness guard cell signaling, and the components regulated by these genes have yet to be identified.

NO and H_2_O_2_ are famous signaling molecules in plants, mediating various important physiological processes, including stomatal movement [7,18,19,20,21,22,23]. They have been reported to contribute to ABA-, salicylic acid-, dark-, UV-B-, and other elicitor-induced stomatal closure [13,19,20,21]. Usually, H_2_O_2_ accumulation acts upstream of NO production in guard cells during stomatal closing [13,19,20,21]. COP1, H_2_O_2_ and NO are all signaling components in guard cells, but little information is available on the relationship of COP1 with H_2_O_2_ and NO during stomatal closing.

In this study, we focused on the role of COP1 in dark-induced stomatal closure and its underlying mechanisms. Through analysis of stomatal phenotypes in *cop1* and *hy5* mutants, we showed that COP1 mediates dark-induced stomatal closure in Arabidopsis, and this process is independent of the transcription factor HY5. Stomatal responses of *cop1* to exogenous H_2_O_2_ or NO, together with the confocal images of endogenous H_2_O_2_ or NO levels in guard cells, further demonstrated that COP1 functions downstream of H_2_O_2_ and upstream of NO during dark-induced stomatal closing. Interestingly, expression of the floral integrator genes *FT*, *TSF* and *SOC1* decreased with dark duration time, and COP1 was required for this regulation. Roles of FT, TSF and SOC1 in dark-induced stomatal closure were further proved by analyzing phenotypes of their corresponding loss-of-function mutants. Finally, the relationship of floral integrators with H_2_O_2_ and NO in darkness were investigated, and the scientific importance of downregulation of *FT*, *TSF* and *SOC1* by COP1 to close stomata normally in the dark was discussed. Our findings provide new insights into the underlying mechanisms of dark-induced stomatal closure.

## 2. Results

### 2.1. COP1 Is Required for Dark-Induced Stomatal Closure

**Figure 1:**
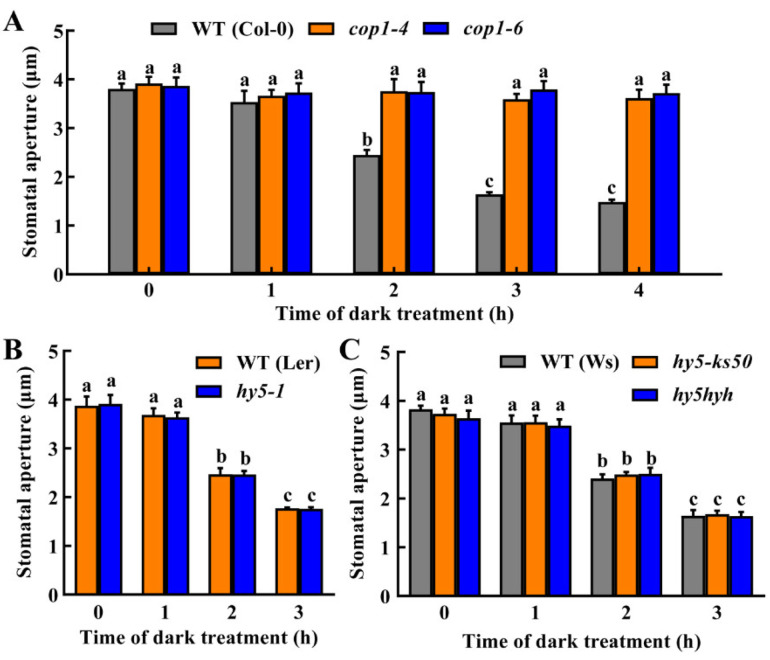
Constitutively Photomorphogenic 1 (COP1) is required for dark-induced stomatal closure in a way independent of long hypocotyl 5 (HY5). (**A**) Stomata of *cop1* mutants were constitutively open in darkness. (**B**,**C**) Mutation of *HY5* does not affect dark-induced stomatal closure. Leaves of WT Col-0, L*er* and Ws, and the *cop1-4*, *cop1-6*, *hy5-1*, *hy5-ks50* and *hy5hyh* mutants were incubated in MES buffer under darkness for 3–4 h. The stomatal apertures of epidermal peels taken from the leaf abaxial surfaces were examined at the indicated time. Data are means ± standard error (SE, n = 3). Different small letters indicate significant differences (*p* < 0.01).

Previous studies have demonstrated that stomata of the *cop1-4* mutant are constitutively open in darkness [6], indicating an important role of COP1 in dark-induced stomatal closure. To confirm the function of COP1, we compared the stomatal responses of *cop1* mutants with that of the wild-type (WT) plants to continuous darkness for 4 h. Results showed that the stomatal aperture of WT plants decreased with the time of dark treatment. A significant decrease was observed at 2 h, and the minimum stomatal aperture appeared at 3 h of dark treatment. Two independent *cop1* mutants showed a constitutive open-stomata phenotype in darkness (Figure 1A), proving the essential role of COP1 in mediating dark-induced stomatal closure.

We previously reported that COP1 mediated UV-B-induced stomatal closure through an HY5-dependent pathway [13]. To determine whether HY5 is also involved in COP1-mediated dark-induced stomatal closure, we compared stomatal responses to the darkness of *hy5* single mutants (*hy5-1* in L*er* background and *hy5-ks50* in Ws background) and *hy5hyh* double mutant with that of the corresponding WT plants. Results showed that mutants for *HY5* had the same stomatal responses to darkness as WT (Figure 1B,C). These results, combined with the facts that HY5 is degraded by COP1 in darkness [8] and that overexpression of HY5 results in constitutive closing of stomata under either light or darkness [13], indicate that HY5 is not involved in dark-induced stomatal closure. Therefore, COP1 mediates dark-induced stomatal closure in a way independent of HY5.

### 2.2. COP1 Acts Downstream of H_2_O_2_ in Dark-Induced Stomatal Closure

**Figure 2:**
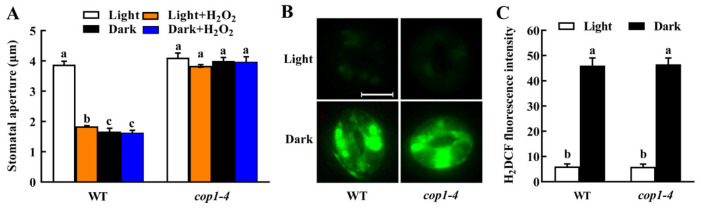
COP1 functions downstream of hydrogen peroxide (H_2_O_2_) in dark-induced stomatal closure. Leaves of WT Col-0 and *cop1-4* mutant were incubated in MES buffer alone or with 100 µmol/L H_2_O_2_ under light or dark for 3 h. (**A**) Exogenous H_2_O_2_ induced stomatal closure in WT Arabidopsis under light but showed no effect on stomatal apertures of *cop1* mutant under light or darkness. (**B**,**C**) Guard cell H_2_O_2_ accumulation in *cop1-4* mutant is similar to that in WT under both light and dark. Images (**B**) of guard cells preloaded with H_2_DCFDA were taken, and fluorescent intensities (**C**) were measured based on the images. Scale bar: 10 µm. Data are means ± standard error (SE, n = 3). Different small letters in (**A**) or (**C**) indicate significant differences (*p* < 0.01).

H_2_O_2_ is a common signal for dark-, ABA- and UV-B-induced stomatal closure [7,13,22]. To explore the signaling pathway in dark-induced stomatal closure, we first investigated the stomatal responses of *cop1* mutant to exogenous H_2_O_2_ treatment. The *cop1* mutant showed a similar stomatal aperture to WT plants under a light. Exogenous H_2_O_2_ significantly closed the stomata of WT but not that of the *cop1* mutant. When transferred to the dark for 3 h, the stomata of the *cop1* mutant are still kept open and exogenous H_2_O_2_ cannot affect this constitutive open-stomata phenotype (Figure 2A). These results indicate that COP1 may function downstream of H_2_O_2_ during dark-induced stomatal closure. To further confirm this, we detected the H_2_O_2_ content in guard cells of *cop1* mutant using a fluorescent dye 2′,7′-dichlorofluorescin diacetate (H_2_DCFDA). The result showed that H_2_O_2_ contents in guard cells of both WT plants and *cop1* mutant were very low under light and increased by several folds in response to 3 h of dark treatment (Figure 2B,C). Clearly, mutation of *COP1* did not affect dark-induced H_2_O_2_ accumulation; however, in contrast to WT plants, the H_2_O_2_ accumulation in the *cop1* mutant did not lead to stomatal closure. This result further confirms that both H_2_O_2_ and COP1 are involved in dark signaling in guard cells, and COP1 is required for transducing the H_2_O_2_ signal.

### 2.3. COP1 Acts Upstream of NO in Dark-Induced Stomatal Closure

**Figure 3:**
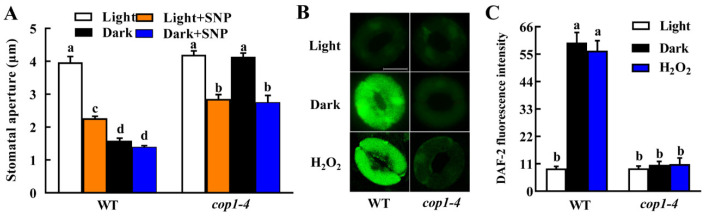
COP1 functions upstream of nitric oxide (NO) in dark-induced stomatal closure. Leaves of WT Col-0 and *cop1-4* mutant were incubated in MES buffer alone or with 50 µmol/L sodium nitroprusside (SNP) under light, dark or 100 µmol/L H_2_O_2_ treatment for 3 h. (**A**) SNP-induced stomatal closure under light and rescued the defect of dark-induced stomatal closure in the *cop1-4* mutant. (**B**,**C**) Dark- and H_2_O_2_-induced NO accumulation in WT were abolished in the *cop1-4* mutant. Images (**B**) of guard cells preloaded with DAF-2DA were taken, and fluorescent intensities (**C**) were measured based on the images. Scale bar: 10 μm. Data are means ± standard error (SE, n = 3). Different small letters in (**A**) or (**C**) indicate significant differences (*p* < 0.01).

NO is another important signal molecule, and NO generation in guard cells during stomatal closure is usually mediated by H_2_O_2_ [19,20,23]. Therefore, both NO and COP1 function downstream of H_2_O_2_. To further define the relationship between NO and COP1 in dark-induced stomatal closure, we investigated the effect of exogenous NO, applied in the form of the NO donor sodium nitroprusside (SNP), on stomatal apertures of *cop1* mutant. SNP treatment significantly reduced the stomatal aperture of WT plants under light conditions and further decreased it under dark treatment (Figure 3A). Meanwhile, SNP treatment not only induced stomatal closure of the *cop1* mutant under light conditions but also significantly rescued the defect of dark-induced stomatal closure in the *cop1* mutant (Figure 3A). These results suggest that COP1 may induce stomatal closure upstream of NO in darkness. Using NO-specific fluorescent dye 4,5-diaminofluorescein diacetate (DAF-2DA), we found that both dark and exogenous H_2_O_2_ treatment significantly enhanced NO accumulation in WT guard cells but did not do that in *cop1* guard cells (Figure 3B,C). This result indicates that dark- and H_2_O_2_-induced NO accumulation are blocked in *cop1* mutant, further confirming that NO accumulation is downstream of COP1 in the dark. However, the stomatal aperture in the *cop1* mutant was significantly larger than that in WT plants under SNP treatment (Figure 3A), indicating that COP1 also mediates dark-induced stomatal closure through alternative pathways.

### 2.4. Expression of FT, TSF and SOC1 Is Downregulated by Darkness via the COP1-Dependent Manner

**Figure 4:**
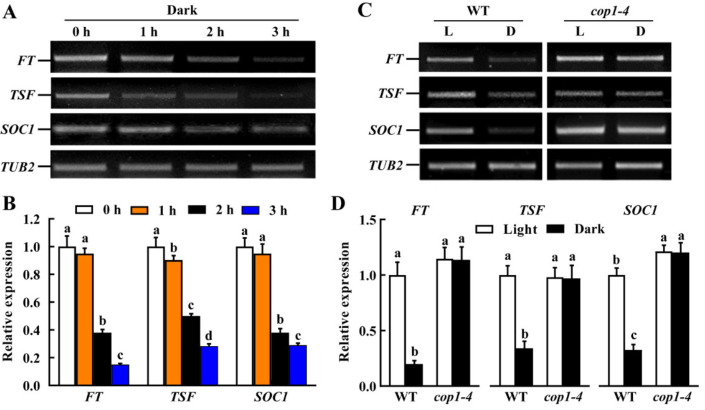
Expression of floral integrator genes in WT and *cop1* mutant in response to dark. (**A**,**B**) Gene expression of *FLOWERING LOCUS T* (*FT*), *TWIN SISTER OF FT* (*TSF*) and *SUPPRESSOR OF OVEREXPRESSION OF CONSTANS1* (*SOC1*) in epidermal peels of WT was downregulated in darkness. Leaves of WT Col-0 were incubated in dark for 0–3 h. (**C**,**D**) Dark-induced downregulation of *FT*, *TSF* and *SOC1* was inhibited by mutation of *COP1*. Leaves of WT Col-0 and *cop1-4* mutant were incubated in MES buffer under light (L) or dark (**D**) for 3 h. Total RNA of epidermal peels were extracted. RT-PCR (**A**,**C**) and qRT-PCR (**B**,**D**) analysis were performed using *TUBLIN BETA CHAIN2* (*TUB2*) as a control. Different small letters indicate significant differences (*p* < 0.01).

The floral integrators FT, TSF and SOC1, have been shown to express in guard cells and positively regulate light-induced stomatal opening [5,16,17]. We performed both RT-PCR and qRT-PCR analysis, and they consistently showed that gene expression of *FT*, *TSF* and *SOC1* in epidermal peels of WT Arabidopsis leaves was downregulated in darkness, and the longer the dark duration time, the lower transcription of these genes was observed during the whole experiment period (Figure 4A,B). Changes in their expression were in agreement with the stomatal responses of WT plants to dark treatment, suggesting a positive correlation between dark-induced stomatal closure and the decreased gene expression of these floral integrators.

To test whether COP1 functions in downregulating the expression of *FT*, *TSF* and *SOC1* in guard cells in darkness, we investigated the effects of *COP1* mutation on the expression of *FT*, *TSF* and *SOC1* in epidermal peels. Results showed that mutation of *COP1* totally inhibited dark-triggered downregulation of these floral integrator genes (Figure 4C,D). These results indicate that the decrease in *FT*, *TSF* and *SOC1* expression are mainly dependent on COP1, and they, in turn, transduce COP1-mediated guard cell signaling in darkness.

### 2.5. FT, TSF and SOC1 Participate in Dark-Induced Stomatal Closure

**Figure 5:**
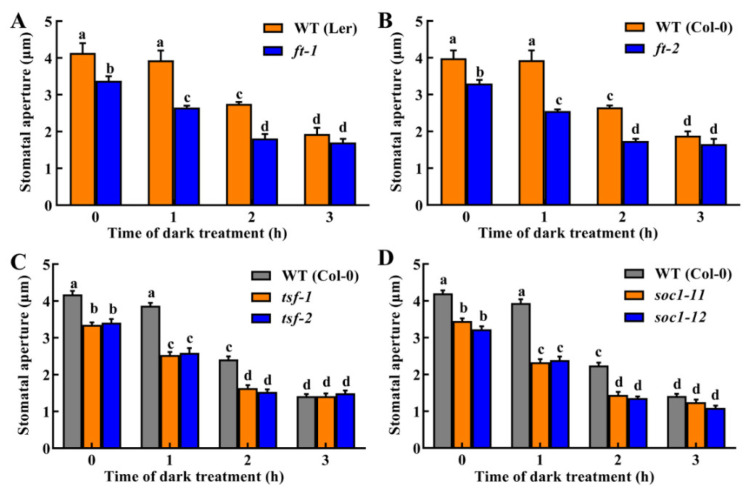
Stomatal responses of *ft*, *soc1* and *tsf* mutants to dark treatment. Leaves of WT Col-0 and L*er*, and the *ft* (*ft-1*, (**A**) and *ft-2*, (**B**)), *soc1* (*soc1-11* and *soc1-12*, (**C**)) and *tsf* (*tsf-1* and *tsf-2*, (**D**)) mutants were incubated in MES buffer in darkness for 0–3 h. The stomata apertures in epidermal peels were examined at 1-h intervals. Data are means ± standard error (SE, n = 3). Different small letters indicate significant differences (*p* < 0.01).

To confirm the role of FT, TSF and SOC1 in dark-induced stomatal closure, we observed the stomatal responses of *ft*, *tsf* and *soc1* mutants to dark treatment. Under light condition (0 h), stomatal apertures of *ft-1*, *ft-2*, *tsf-1*, *tsf-2*, *soc1-11* and *soc1-12* mutants were all significantly smaller than that of WT controls (Figure 5), confirming light-induced stomatal opening is suppressed in these mutants [5,16,17]. Under darkness for 1 h, the stomatal aperture in WT plants showed a slight but not significant decrease, while it significantly decreased in *ft*, *tsf* and *soc1* mutants. Under darkness for 2 h, both WT and mutants closed their stomata further. However, by comparing stomatal apertures, we found that *ft*, *tsf* and *soc1* mutants maximumly closed their stomata at 2 h (Figure 5), while WT did it at 3 h (Figure 1 and Figure 5). These results demonstrate the hypersensitive stomatal responses of *ft*, *tsf* and *soc1* mutants to darkness, indicating that FT, TSF and SOC1 not only positively regulate light-induced stomatal opening but also negatively modulate dark-induced stomatal closing.

### 2.6. Mutation of FT, TSF and SOC1 Accelerates Dark-Induced NO but Not H_2_O_2_ Accumulation in Guard Cells

**Figure 6:**
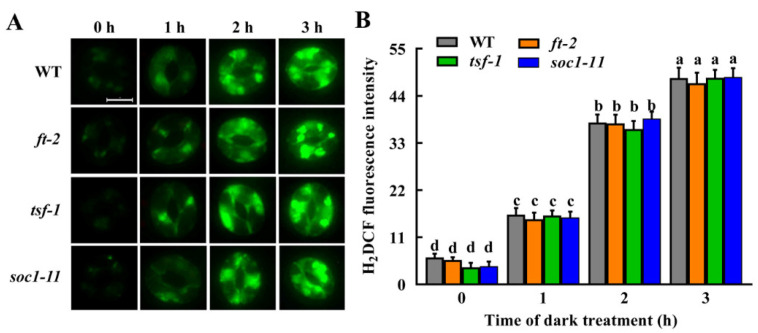
Mutation of *FT*, *TSF* or *SOC1* does not affect dark-induced H_2_O_2_ accumulation in guard cells. Leaves of WT Col-0 as well as *ft-2*, *tsf-1* and *soc1-11* mutants, were incubated in MES buffer under darkness for the indicated time. Then the images (**A**) and fluorescence intensities (**B**) of guard cells preloaded with H_2_DCFDA were recorded. For (**A**), scale bar = 10 μm. For (**B**), data are means ± standard error (SE, n = 3). Means with different letters are significantly different at *p* < 0.01.

Given that FT, TSF and SOC1, as well as H_2_O_2_ and NO, all participate in dark-induced stomatal closure, we further clarified the relationship of H_2_O_2_ or NO with the floral integrators FT, TSF and SOC1 by monitoring H_2_O_2_ and NO levels in guard cells of *ft*, *tsf* and *soc1* mutants. We found that *ft*, *tsf* and *soc1* mutants showed the same level of H_2_O_2_ as the WT plants under light or during the dark treatment for 1–3 h (Figure 6), indicating that mutation of *FT*, *TSF* and *SOC1* do not affect dark-induced H_2_O_2_ accumulation.

**Figure 7:**
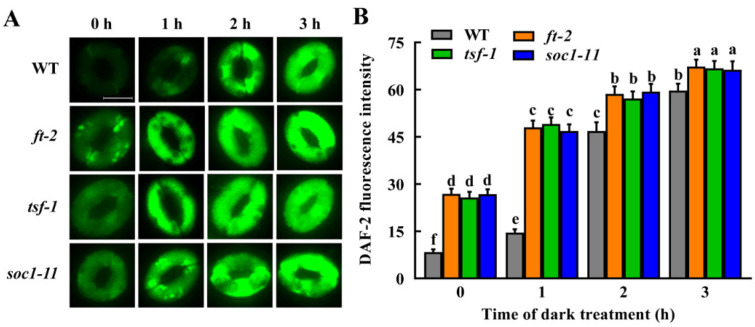
Mutation of *FT*, *TSF* or *SOC1* facilitates dark-induced NO accumulation in guard cells. Leaves of WT Col-0 and *ft-2*, *tsf-1* and *soc1-11* mutants were incubated in an MES buffer under darkness for the indicated time. Then the images (**A**) and fluorescence intensities (**B**) of guard cells preloaded with DAF-2DA were recorded. For (**A**), scale bar = 10 μm. For (**B**), data are means ± standard error (SE, n = 3). Means with different letters are significantly different at *p* < 0.01.

In contrast, the NO level in the guard cells of these mutants was significantly higher than that of WT plants under light conditions and increased faster than that of WT in response to dark treatment (Figure 7). These results indicate that mutation of *FT*, *TSF* and *SOC1* accelerates guard cell NO accumulation under either light or darkness. Obviously, changes in NO level have a significantly negative correlation with the stomatal apertures in these mutants under light and dark treatment (*r* = −0.849, *p* = 0.008 for *ft-2*; *r* = −0.891, *p* = 0.003 for *tsf-1*; and *r* = −0.890, *p* = 0.003 for *soc1-11*), suggesting that downregulation of *FT*, *TSF* and *SOC1* expression by darkness facilitates NO accumulation in guard cells and therefore contributes to dark-induced stomatal closure.

## 3. Discussion

### 3.1. COP1 Mediates Dark-Induced Stomatal Closure through Promotion of NO Accumulation

The E3 ubiquitin ligase COP1 has been well-documented as a central, negative determinant for plant photomorphogenesis [8]. It directs the degradation of diverse light-regulated transcription factors by the 26S proteasome [8,24]. Additionally, it also acts as a negative regulator of stomatal development [25]. Recently, increasing evidence also suggests a crucial role of COP1 in regulating stomatal movement [2,9,10,11,12,13]. These studies demonstrate that COP1 participates in both light-induced stomatal opening and ABA- and UV-B-induced stomatal closure. In addition, COP1 has also been indicated to play a role in dark-induced stomatal closing [6]. Here, the constitutive open-stomata phenotype of both *cop1-4* and *cop1-6* mutants provides further evidence that COP1 is required for dark-induced stomatal closure.

HY5 is one of the most studied signaling transcription factors, which sometimes acts redundantly with its closely related homolog HYH (HY5 HOMOLOG) [20,26]. The interaction between HY5 and COP1 is involved in many developmental processes [26]. In photomorphogenesis, COP1 acts as a negative factor to promote the degradation of HY5 [26]. However, it has been reported that, in UV-B signaling, COP1 positively regulates HY5, and HY5 then mediates UV-B-induced stomatal closure [13,20]. In the present study, neither *hy5* single mutants nor *hy5hyh* double mutants affected stomatal responses to dark treatment (Figure 1), indicating HY5 is not involved in the dark-signaling of guard cells. Our result indicates that, in contrast to UV-B signaling, COP1 acts in a way independent of HY5 during dark-induced stomatal closure.

H_2_O_2_ and NO are famous signaling molecules mediating various physiological processes in plants, including stomatal movement [7,18,19,20,21,22,23,27]. Zhang et al. [21] provided convincing evidence that H_2_O_2_ and NO participate in dark-induced stomatal closure, in which H_2_O_2_ acts upstream of NO. Here, after confirming the essential role of COP1, we further revealed the relationships among COP1, H_2_O_2_ and NO in dark-induced stomatal closure. Pharmacological and confocal studies strongly support that COP1 functions downstream of H_2_O_2_ and upstream of NO in dark-induced stomatal closure (Figure 2 and Figure 3). Similar relationships have also been reported for COP1, H_2_O_2_ and NO in UV-B-induced stomatal closure [13]. Further studies are needed to uncover how COP1 is regulated by H_2_O_2_ and then mediates NO production in UV-B- and dark-induced stomatal closure. Except for COP1, the MEK1-MPK6 cascade also functions in dark-induced stomatal closure by mediating H_2_O_2_-induced NO accumulation [21]. Whether and how COP1 has crosstalk with MEK1-MPK6 cascade is worth to be further studied. AtMYB61, an R2R3-MYB transcription factor specifically expressed in guard cells, plays a major role in dark-induced stomatal closure [28]. Moazzam-Jazi et al. [12] reported that COP1 expression in darkness is required for the expression of AtMYB61. A study on the relationship between AtMYB61 and NO will also provide a better understanding of COP1 signaling in darkness.

### 3.2. Floral Integrators FT, TSF and SOC1 Negatively Regulate Dark-Induced Stomatal Closure

Although FT, TSF and SOC1 were originally identified and further well-studied as crucial flowering regulators, their roles in other physiological processes, such as fruit set and vegetative growth, have also received a lot of attention [14,15]. These researches suggest that they are general growth regulators in plants. In the past 11 years, a novel function of these proteins as regulators of stomatal movement was defined successively [5,16,17,29]. However, these studies focused on light-induced stomatal opening, in which *FT*, *TSF* and *SOC1* expressed highly and functioned as positive regulators. Here, we not only revealed that expressions of *FT*, *TSF* and *SOC1* all decreased with dark duration time (Figure 4) but also provided genetic evidence that these floral integrators are negative regulators in dark-induced stomatal closure (Figure 5). These findings add new information on the role of FT, TSF and SOC1 as stomatal movement regulators. However, the partially closed phenotypes of *ft*, *tsf* and *soc1* mutants under light suggest that alternative signaling pathway(s) independent of *FT*, *TSF* and *SOC1* also exist in dark-induced stomatal closure, which should be further studied.

### 3.3. COP1 Promotes NO Accumulation by Suppressing FT, TSF and SOC1 Expression

The decreased expression levels of *FT*, *TSF* and *SOC1,* were positively correlated with the gradually reduced stomatal aperture during dark treatment (Figure 4A). This result is consistent with previous results that stomatal opening was accompanied by the up-regulation of *FT*, *TSF* and *SOC1* expression [5,16,17]. Together, these results demonstrate that the regulation of dark-induced stomatal closure by FT, TSF and SOC1 is due to the downregulation of their expressions. Moreover, *COP1* mutation not only blocked the downregulation of *FT*, *TSF* and *SOC1* expression in darkness (Figure 4B) but also impaired dark-induced stomatal closure (Figure 1). These results indicate that COP1 mediates dark-induced stomatal closure by suppressing *FT*, *TSF* and *SOC1* expression. CO, a major regulator of photoperiodic flowering response, is also involved in stomatal opening [5]. It is well-known that *FT* and *TSF* are targets of CO protein, and *SOC1* expression is mediated by FT [5,29]. Besides, we also know that COP1 targets CO for proteasome-mediated degradation in the dark [8,24]. Therefore, COP1 mediates the down-regulation of *FT*, *TSF* and *SOC1* expression in darkness by promoting the degradation of CO, the upstream regulator of *FT*, *TSF* and *SOC1*. Further studies are needed to test this hypothesis.

To date, little information is available on the relationship of signaling molecules H_2_O_2_ and NO with floral integrators FT, TSF and SOC1, especially in guard cells. In the present study, we showed that H_2_O_2_ accumulation in guard cells of *ft*, *tsf* and *soc1* mutants is similar to that of WT plants (Figure 6), while NO in guard cells of *ft*, *tsf* and *soc1* mutants accumulated faster and higher than that of WT plants under dark treatment (Figure 7). This result, together with the stomatal phenotypes, indicates that decreased expression of *FT*, *TSF* and *SOC1* does not affect H_2_O_2_ but promotes NO accumulation in guard cells and consequently facilitates dark-induced stomatal closure. Our findings enrich mechanisms of guard cell signaling in darkness and also pose an interesting challenge in elucidating the molecular mechanisms underlying FT-, TSF-, and SOC1-mediated stomatal movement.

It has been recognized that an appropriately functioning circadian system confers an adaptive advantage to plants [30]. Wang et al. [31] demonstrated that manipulating H^+^-ATPase in guard cells to make stomata open wider in the daytime and still close at night enhanced plant growth while making the stomata constantly open, even in the dark, did not. As major regulatory components were mediating the onset of flowering, expression of the floral integrators (FT, TSF and SOC1) will be activated prior to flowering time [15]. These photoperiodic flowering components positively affect light-regulated stomatal opening, probably resulting in the acceleration of photosynthesis to prepare for flowering [5]. The improved growth may be offset by increased water loss if stomata are still open in the dark [30]. Therefore, our finding that downregulation of *FT*, *TSF* and *SOC1* by COP1 to allow stomata to close normally in the dark may be an important mechanism facilitating plant growth, especially in the period prior to the flowering time when the floral integrators are highly expressed.

**Figure 8:**
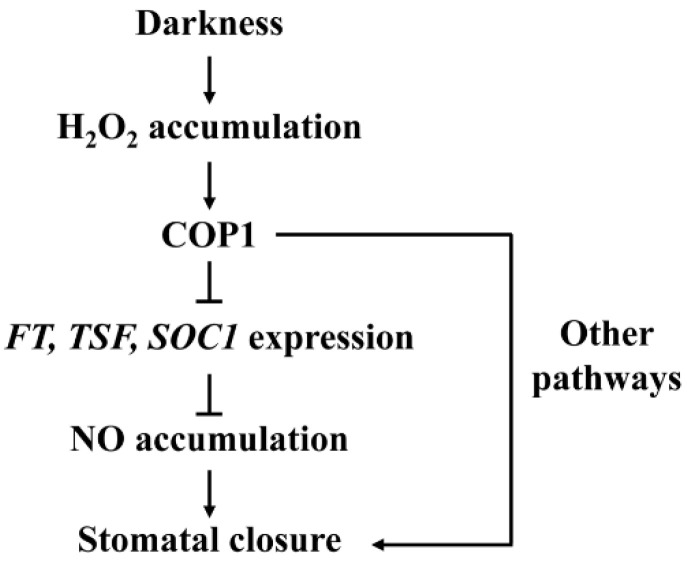
A possible model of COP1-mediated dark-induced stomatal closure. Arrows and bars indicate positive and negative regulation, respectively.

In summary, the data presented herein demonstrate that COP1 acts downstream of H_2_O_2_ to mediate dark-induced NO accumulation in guard cells and subsequent stomatal closure by suppressing expressions of the floral integrator genes *FT*, *TSF* and *SOC1*. Based on our findings, we put forward a possible model by which COP1 mediates dark-induced stomatal closure (Figure 8). The novel role of *FT*, *TSF* and *SOC1* as negative regulators of dark-induced stomatal closure and the interrelationships among H_2_O_2_, COP1, *FT*/*TSF*/*SOC1*, and NO would no doubt provide clues directing a further study on guard cell signal transduction.

## 4. Materials and Methods

### 4.1. Plant Materials and Growth Conditions

The following Arabidopsis (*Arabidopsis thaliana*) lines were used in this study: Col-0, L*er*, Ws, the COP1 mutants *cop1-4* and *cop1-6* [13], HY5 mutants *hy5-1* and *hy5-ks50* [13], *hy5hyh* double mutant [20], FT mutants *ft-1* and *ft-2* [16], TSF mutants *tsf-1* [5] and *tsf-2* (SALK_064104), and the SOC1 mutants *soc1-11* (SALK_006054) and *soc1-12* (SALK_138131). *hy5-1* and *ft-1* were derived from L*er* background, *hy5-ks50* and *hy5hyh* from Ws background, and all other mutants from Col-0 background. *cop1-4* and *hy5hyh* mutants were kindly provided by Dr. G.I. Jenkins (University of Glasgow, UK) and Dr. X.W. Deng (Yale University, New Haven, CT, USA), respectively. Other seeds were purchased from the Nottingham Arabidopsis Stock Center (NASC, Nottingham, UK). All mutants were confirmed by PCR analysis.

Seeds of wild types and mutants were surface sterilized with bleaching power (20%, *w*/*v*) for 10 min, washed with sterilized water three times, then germinated and grown on vermiculite. Seedlings were cultured as previously described [21]. Fully expanded leaves of four–five-week-old healthy plants were harvested and immediately used for different treatments.

### 4.2. Stomatal Bioassay

The stomatal bioassays were performed according to Zhang et al. [21] using the rosette leaves of four–five-week-old WT plants or tested mutants with slight modifications. Freshly detached leaves were incubated in MES-KCl buffer (50 mmol/L KCl, 10 mmol/L MES-KOH, pH 6.15) under white light (PPFD 0.1 mmol/m^2^/s) for 2 h to open the stomata, and then incubated in the buffer alone or with 100 μmol/L H_2_O_2_ or 50 μmol/L SNP under the same white light or darkness for 1–4 h. After treatments, abaxial epidermal strips were peeled from the leaves, and stomatal apertures were measured with a calibrated light microscope. Stomatal bioassays were always started at the same time of the day to avoid the potential rhythmic effects on stomatal aperture. Data are displayed as means ± standard errors (SE) of three biological replicates, each with 40 stomata.

### 4.3. Measurement of Endogenous H_2_O_2_ and NO in Guard Cells

Endogenous H_2_O_2_ and NO in guard cells were measured according to a previous study [21] with fluorescent dye H_2_DCFDA and DAF-2DA, respectively. After being incubated with the fluorescent dye and removing the excess dye in the dark, fluorescence of the epidermal strips was immediately observed using a confocal laser scanning microscopy (Leica TCS-SP8 Lasertechnik GmbH, Wien, Austria, excitation 488 nm, emission 515 ± 15 nm, frame 1024 × 1024). Images were then processed with Photoshop software, and the whole stomata areas were analyzed with Leica Image software to quantify the fluorescence intensity. All presented images represented similar results from three independent experiments, and the data of fluorescence intensity are presented as means ± SE of three independent experiments, each with 20 stomata.

### 4.4. Gene Expression Analysis

Total RNAs were extracted from the epidermal peels with E.Z.N.A.™ Plant RNA Kit (OMEGA, La Chaux-de-Fonds, Switzerland), and first-strand cDNAs were synthesized using the PrimeScript™ RT Master Mix (TAKARA, Shiga, Japan) following the manufacturer’s instructions. *TUBELIN BETA CHAIN2* (*TUB2*) was used as the control. Twenty-five cycles of PCR were performed for *FT*, *TSF*, *SOC1* and *TUB2* in RT-PCR. Primer sequences for RT-PCR analysis of these genes were reported previously [17]. Quantitative gene expression was analyzed by qRT-PCR with SYBR Green PCR Master Mix as previously described [32]. The primers that have been successfully used for *FT*, *SOC1*, *TUB2* [29] and *TSF* [33] were used in this study for qRT-PCR analysis.

### 4.5. Statistical Analysis

All experiments were repeated three times with similar results. Statistical analysis was performed using a one-way ANOVA to discriminate significant differences followed by least significant difference test (LSD).

## Data Availability

Data will be made available upon request.
